# A Rare Case of Neurosyphilis with Ocular Involvement in a Patient with HIV Infection and New Onset Syphilis

**DOI:** 10.7759/cureus.4034

**Published:** 2019-02-07

**Authors:** Sandeep Koripalli, Libardo Rueda Prada, Padma Priya P Gummadi, Shorabh Sharma, Kaniz Banu

**Affiliations:** 1 Internal Medicine, St. Barnabas Hospital, Bronx, USA; 2 Internal Medicine, Creighton University School of Medicine, Omaha, USA

**Keywords:** sexually transmitted diseases, spirochaetales infections, treponemal infections, central nervous system

## Abstract

Neurosyphilis (NS) is more frequently seen in patients with human immunodeficiency virus (HIV) infection, especially those not on antiretroviral therapy or with a low CD4 cell count. Ocular syphilis is an unusual and early form of neurosyphilis. Lumbar puncture should be considered in all HIV infected patients who present with neurologic or ocular disease. A 47-year-old homosexual male with HIV-1 infection, on antiretroviral therapy (last CD4 cell count 1022 cells/μL) presented to our emergency department with a five-day history of headache, blurry vision, pain and redness of the left eye. He had unprotected anal sex with a new partner four months before presentation. Based on the fundoscopy findings as well as the cerebrospinal fluid (CSF) analysis on initial evaluation, a repeat serum rapid plasma reagin (RPR) along with microhemagglutination assay for treponema pallidum (MHA-TP) were done due to high suspicion of syphilis, even though an RPR five months prior to this visit was negative. Both RPR and MHA-TP were positive and the patient was treated for neurosyphilis. The patient’s symptoms as well as the RPR titers improved significantly thereafter. A high index of suspicion for neurosyphilis should be maintained in HIV-infected patients presenting with ocular symptoms even if they are compliant with retroviral therapy with good CD4 cell counts. Physicians must be mindful of this uncommon presentation and consider a lumbar puncture in any patient with suspicion of neurosyphilis for prompt diagnosis and treatment to avoid further neurological complications.

## Introduction

Syphilis is a sexually transmitted disease caused by the spirochete Treponema pallidum. It has a higher incidence in human immunodeficiency virus (HIV) positive patients, being most common in men who have sex with men [1]. Acquired syphilis is classified into early syphilis (primary, secondary and early latent), late syphilis, and neurosyphilis (NS). NS is the infection of the central nervous system (CNS) identified at any stage and is more frequently seen in patients with HIV infection, especially those not on antiretroviral therapy or with a low CD4 cell count. Ocular syphilis is an unusual, rare presentation and an early form of neurosyphilis.

## Case presentation

A 47-year-old homosexual male presented to the emergency room with a five-day history of intermittent frontal and retro-orbital headache, progressive blurriness of vision, and photophobia associated with redness, excessive watering and pain in his left eye. Two weeks before presentation, he developed left knee swelling and pain accompanied by a non-specific skin rash, which resolved spontaneously within two to three days. His past medical history was remarkable for chronic kidney disease stage II and HIV-1 infection with a latest CD4 count of 1022 cells/mm3. The patient was allergic to sulfa drugs. He was compliant with his antiretroviral therapy, which included dolutegravir, darunavir, tenofovir, emtricitabine, and ritonavir with no renal dose adjustments required as creatinine clearance (CrCl) was > 60 mL/min. The patient had unprotected anal intercourse with a new partner four months as well as one month prior to this admission.

**Figure 1:**
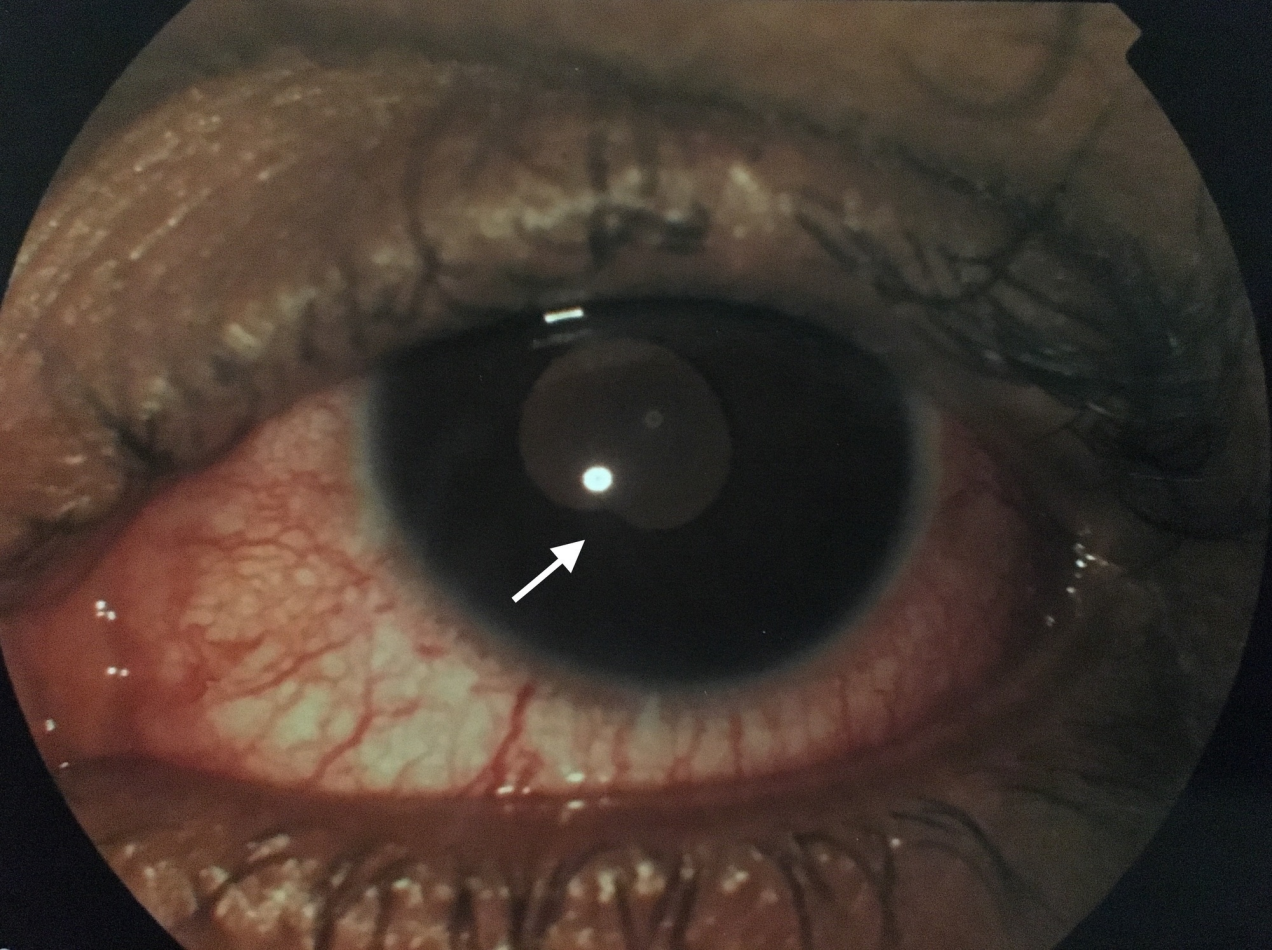
Left eye exam. Conjunctival injection with cells in the anterior chamber and posterior synechiae at 7 O’ clock position (white arrow).

**Figure 2:**
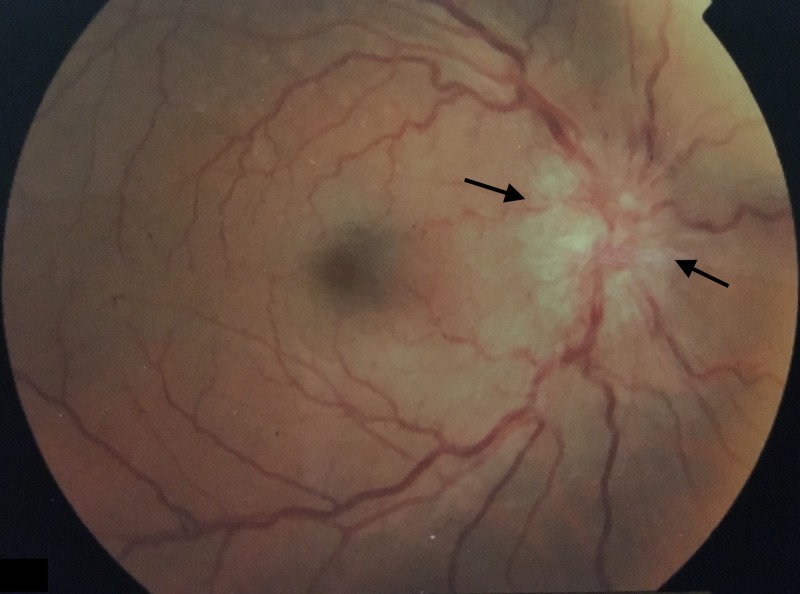
Left eye indirect ophthalmoscopy. Papilledema with blurred disk margins (black arrows).

On physical examination, the patient was in non-acute distress, alert, and fully oriented; other vitals signs were as follows: afebrile, heart rate of 91 bpm, blood pressure 126/80 mmHg, respiratory rate 18 rpm, and oxygen saturation 100% at room air. An ophthalmologic examination revealed bilateral visual acuity of 20/70. The pupils were equally round and reactive to light; there was no relative afferent pupillary defect. A slit-lamp examination revealed in the left eye 2+ injection of the conjunctiva, 3+ cells in the anterior chamber and posterior synechiae at 7 O’ clock position (Figure 1). Indirect ophthalmoscopy revealed +1 cells in the left vitreous, blurred posterior margins bilaterally with cup-to-disk ratio of 0.1, consistent with papilledema (Figure 2). In short, the patient had left eye uveitis and bilateral papilledema. There were no meningeal signs or neurological signs of focalization. There were no additional physical exam findings.

Initial workup revealed creatinine at baseline level, normal platelet, red and white blood cell counts. Initial imaging studies, computed tomography (CT) and magnetic resonance imaging (MRI) of the brain showed no abnormalities. A lumbar puncture was performed and the patient was started on empiric treatment for meningitis with ceftriaxone 2 grams every 12 hours, vancomycin 1000 mg every 12 hours, ampicillin 2 grams every four hours, acyclovir 800 mg every eight hours, and dexamethasone in order to cover for the most typical pathogens such as Streptococcus pneumoniae, Neisseria meningitidis, Listeria monocytogenes, aerobic Gram negative bacilli and Herpes simplex virus. Doxycycline 100 mg every 12 hours was added to the regimen as well due to suspicion of Reiter’s syndrome given the uveitis and joint involvement. His outpatient anti-retroviral therapy was continued. Cerebrospinal fluid (CSF) analysis revealed 45 cells/μL white blood cells, 2% being neutrophils, 94% lymphocytes, 4% monocytes; RBC of 80 cells/mm3; glucose of 49 mg/dL; and protein of 126 mg/dL suggestive of lymphocytic meningitis. Further, the results revealed that his CSF was negative for Herpes simplex virus 1 and 2, Cryptococcus, Epstein-Barr Virus (EBV); the Venereal Disease Research Laboratory (VDRL) test was non-reactive and no organisms were detected on initial Gram stain. A blood workup revealed an absolute CD4 count of 1022 cells/mm3, a rapid plasma reagin (RPR) test reactive with a titer of 1:128, and a positive microhemagglutination test for antibodies to Treponema pallidum (MHA-TP). The RPR test had been negative five months prior to this admission during a routine outpatient clinic visit. Gonococcal and chlamydial cultures on nasal, throat, and anal swabs were negative.

Treatment was initiated with intravenous penicillin G, 24 million units/day given as 4 million units every four hours for 14 days and cyclopentolate, prednisone drops for the affected eye. During the hospital course, the patient’s ocular symptoms including blurriness of vision and redness improved within two to three days and he was discharged home. On follow-up at the Infectious Disease Clinic, the patient was asymptomatic with constantly decreasing RPR titers from 1:128 at the time of admission to 1:16 at three months and 1:8 at six months from admission. Follow-up at the ophthalmology clinic showed no evidence of uveitis or papilledema. The patient refused a repeat lumbar puncture on his visit after six months.

## Discussion

In the pre-antibiotic era NS was common, occurring in 34% of patients with syphilis [2]. In the current era, HIV-infected patients are more prone to develop NS, with higher risk in patients with CD4 cell counts ⩽350 cells/μL and RPR titer >1:32 [3, 4]. Ocular involvement is very rare in NS and accounts for only 1%-5% of the cases in the United States [5,6].

The early form of NS typically affects the CSF, meninges, and vasculature, while the late forms affect the brain and spinal cord parenchyma. Patients with early NS can be asymptomatic with positive CSF serology or can develop symptomatic meningitis. These patients may develop spontaneous resolution without mounting an inflammatory response or may develop transient meningitis before resolution. Persistent meningitis may develop if the organism cannot be cleared from the CSF and these patients are initially asymptomatic but can develop symptoms anytime. Patients with symptomatic syphilitic meningitis might complain of headache, confusion, nausea and vomiting, and stiff neck [7]. Visual acuity may be impaired if there is concomitant uveitis, vitritis, retinitis, or optic neuropathy.

The first step in establishing the diagnosis of NS is to confirm the infection. A thorough neurological exam is suggested in all patients with HIV infection and syphilis of any stage. Like in the above discussed case, a small clue of ocular or central nervous system manifestations should prompt a lumbar puncture to be performed on the patient. The diagnosis is based on the identification of CSF abnormalities, including a lymphocytic pleocytosis that is typically <100 cells/μL, an elevated protein concentration that is usually <100 mg/dL, a reactive CSF-VDRL, or a combination of these abnormalities. Serum studies can also support the diagnosis of NS in patients with high clinical suspicion of the disease with indeterminate CSF analyses results. In late latent syphilis, the non-treponemal tests (VDRL and RPR) may be nonreactive [8,9]. In the context of a negative non-treponemal test in a patient highly suspicious for syphilis, the prozone phenomenon must also be kept in mind, before relying on the negative test results [10]. When there is suspicion for late forms of NS, serum treponemal tests such as fluoroscent treponemal antibody absorption (FTA-ABS) should always be performed [11]. These tests remain reactive for life in virtually all individuals regardless of previous treatment. In patients with NS who have HIV infection with CSF pleocytosis but nonreactive CSF-VDRL, it is difficult to establish a definite diagnosis because mild CSF pleocytosis and elevated protein can be due to the HIV infection itself [12]. Even though our patient had a negative RPR five months prior to this presentation and a negative VDRL on CSF analysis, a repeat RPR testing was done due to the recent unprotected sex and the CSF lymphocytic pleocytosis, both of which together were highly suspicious for neurosyphilis. Due to the right clinical context and appropriate lab findings, a presumptive diagnosis of NS was made without further tests, such as FTA-ABS, and treatment was begun. As per the Centers for Disease Control and Prevention (CDC) guidelines, sexual partners of patients receiving the diagnosis of primary, secondary or early latent syphilis should be confidentially notified of the exposure and the need for evaluation to be presumptively treated for early syphilis [13].

Ocular syphilis is rarely seen during primary syphilis but may be seen in the secondary stage and, more frequently, in late, latent, and tertiary stage. The most common finding is panuveitis regardless of HIV status. Other findings may include chorioretinitis, anterior uveitis, optic neuropathy, interstitial keratitis, and retinal vasculitis. Delay in a prompt diagnosis may result in poor visual outcomes.

Patients with NS, regardless of symptoms and CSF-VDRL reactivity, should be treated to prevent progression to symptomatic disease. Ocular syphilis, otosyphilis, and neurosyphilis require treatment with a 10-to-14-day course of high-dose intravenous penicillin. A repeat lumbar puncture six months post treatment is necessary to assess response to treatment. Prompt initiation of antibiotic treatment in patients with ocular syphilis is essential to prevent irreversible vision loss. Normal results of a CSF analysis do not rule out ocular syphilis as Treponema pallidum may infect the eye without infecting the brain or meninges. Immediate treatment for ocular syphilis should be initiated regardless of the results of CSF analysis.

## Conclusions

With the incidence of syphilis on the rise, clinicians should have a high index of suspicion for NS during encounters with HIV-infected patients complaining of visual symptoms, as there is rapid progression of the disease in such patients. Relying on the positive CSF-VDRL test is not necessary to diagnose neurosyphilis. Ocular syphilis must be treated as NS regardless of the results of CSF analyses. The risk of progression of ocular syphilis to NS is higher in patients with low CD4 counts. However, patients with good CD4 counts can also rarely progress to NS as seen in this case. An early identification of the infected patients will allow the clinician to perform a prompt diagnosis and start early treatment, which will avoid further complications in such patients.
